# Supplementary material for: Fast custom wavelet analysis technique for single molecule detection and identification
Source: Nat Commun. 2022 Feb 24;13:1035. doi: 10.1038/s41467-022-28703-z (PMC8873225; doi:10.1038/s41467-022-28703-z)
Supplement: Supplementary file 1 — Supplementary Information [file 41467_2022_28703_MOESM1_ESM.pdf]

Supplementary Information for

"Fast custom wavelet analysis technique for single molecule detection and identification"

V. Ganjalizadeh[a], G. G. Meena[a], T. A. Wall[b], M. A. Stott[b], A. R. Hawkins[b], H. Schmidt[a]*

[a]School of Engineering, University of California, Santa Cruz, 1156 High Street, Santa Cruz, California 95064, USA;

[b]Electrical and Computer Engineering Department, Brigham Young University, Provo, Utah 84602, USA

Additional Single-Peak Signal Analysis

Similar to the multi-peak events discussed in the main manuscript, single-peak events detected by the PCWA algorithm for 200 nm fluorescent beads (Supplementary Fig. 1a) contain not only the (temporal) location and magnitude of the peaks, but also the Δt (scale) values. Here, Δt represents the width of the Ricker wavelet that matches the fluorescence peak. This Δt value can be converted to the particle's flow velocity using the known spatial FWHM of the excitation waveguide mode. We visualized the intensity and Δt (and/or velocity) information of all detected events in a joint histogram and the distribution of the events matched well with the kernel predicted from a simulation model that takes into account the distribution of particles and flow velocity profile in the fluidic channel (Supplementary Fig. 1c). Real-time analysis of the data can also help with monitoring multiple metrics of the experiment, i.e. the velocity and event rate to reflect possible clogging of the device. Supplementary Figure 1d illustrates this using the examples of detected events over time (top panel) and time-dependent fluorescence intensity and particle velocity (bottom panel).

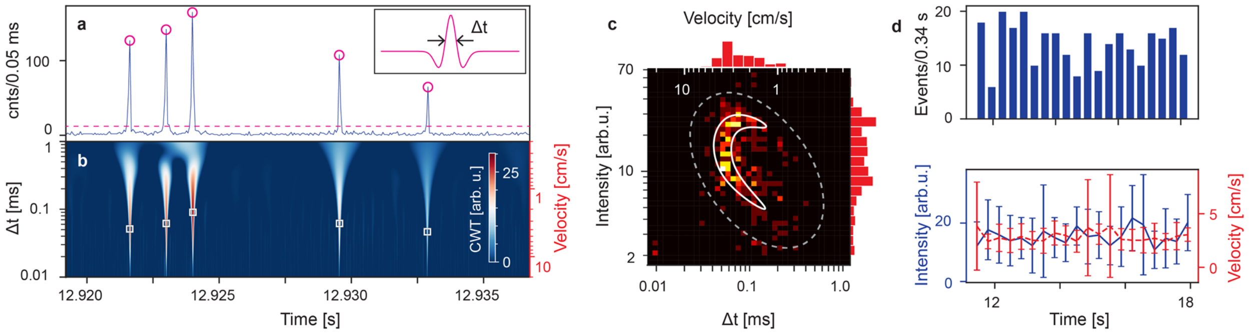

Supplementary Fig. 1 Advanced single-peak signal analysis. **a** Zoomed in window of events from five fluorescent beads. Inset is the Ricker wavelet used to detect the events with Δt parameter set as scaling factor to get velocity information for each event. The dashed line indicates the threshold level used if events are identified by a conventional

peak finding algorithm. **b** CWT coefficient scalogram with detected peaks shown as square boxes. **c** Joint histogram of the intensity and Δt information extracted for individual events. A good match with expected distribution (area contained by solid white line) is observed. **d** Time varying representation of multiple pieces of information for detected peaks gives useful insight about experiment dynamics. Error bars represent standard deviation. No additional post-processing was needed to extract Δt information.

Nanopore Translocation Detection

Nanopore sensors have emerged as ultrasensitive tools for detection and analysis of individual nanoparticles with numerous applications such as next-generation sequencing[1–3]. They operate on the principle that individual particles moving through a nanoscopic membrane generate a characteristic modulation of an ionic current across the membrane. In this way, time-dependent electrical signals due to single events are produced analogous to the optical fluorescence signals discussed in the main manuscript. We evaluated the PCWA algorithm by analyzing electrical signals recorded from the optofluidic chip augmented for use as a nanopore sensor. Supplementary Figure 2a illustrates the experimental setup for the experiment where single SARS-CoV-2 RNAs were driven through the nanopore by an applied voltage V_{NP}. The optofluidic nanopore device shown in Supplementary Fig. 2a consists of a hollow-core waveguide delivering the target molecules, here SARS-CoV-2 RNAs bound to microbeads from inlet reservoir (1) to the nanopore capture region by the applied electrokinetic voltage (V_{EK}). A trap-assisted capture rate enhancement (TACRE) technique[4] employs the optical force from a light beam in the liquid-core waveguide to locally trap multiple beads holding target molecules underneath the nanopore. This increases the target concentration at the

nanopore location and the rate of detection upon thermal release from the beads by orders of magnitude. Translocations of individual released nucleic acid molecules through the nanopore are detectable from the current change between reservoirs (2) and (3). Multiple factors such as shape and location of DNA molecule can impact the blockade time and depth and therefore, significant variations of the blockade events are typically seen in the electrical current signal. The multi-scale new CWT technique can tackle the peak finding challenge here by looking for peaks at different scales to improve detection accuracy significantly. Supplementary Figure 2b-d show how the PCWA algorithm was able to detect the blockade events successfully without any pre-processing of the raw data. The high speed of the PCWA algorithm makes this approach interesting for time-sensitive applications of nanopore sensing such as feedback-controlled, on-demand delivery of DNA molecules[5].

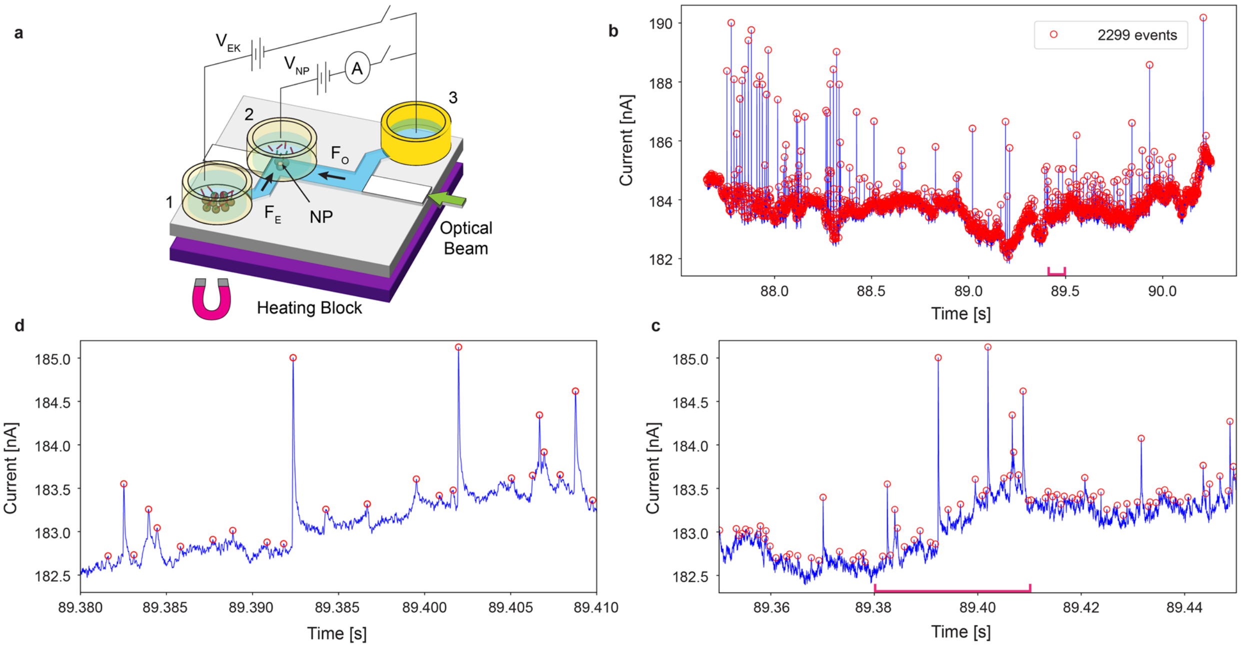

Supplementary Fig. 2 PCWA analysis of single molecule nanopore sensor. **a** Schematic of the experimental setup to detect SARS-CoV-2 nucleic acids. **b** Full trace

with ~2,000 events detected in 2.6 s long trace. **c-d** Zoomed in windows to show location of the detected events using PCWA and two other CWT peak detectors[6,7].

Mass Spectroscopy (MS) Peak Detection

In order to benchmark accuracy and recall performance of the PCWA method, we ran a peak detection task on a simulated mass spectra dataset[8]. This dataset provides simulated protein spectra with noisy raw data alongside the true location of the peaks, which enables the extraction of a receiver operating characteristic (ROC) curve. A ROC curve is a means to visualize and compare the performance of a detector across a range of threshold parameters for the detector. The *x* and *y* axes of a ROC curve correspond to True Positive Rate (TPR or recall) and False Positive Rate (FPR), respectively. A perfect detector would create a single point in the top-left corner of the ROC space (TPR=1, FPR=0), independent from the set threshold. The area under curve (AUC) for a ROC curve is a number between 0 and 1 for normalized FPR and TPR and a higher AUC value means a better detector. Specifically, the True Positive Rate (TPR) and the False Positive Rate (FPR) are calculated as

$$TPR = \frac{TP}{TP+FN}\,, \quad FPR = \frac{FP}{FP+TN}\,, \quad \begin{matrix} TP\text{: } true\ positive, & FP\text{: } false\ positive \\ TN\text{: } true\ negative, & FN\text{: } false\ negative \end{matrix}\,, \qquad (1)$$

In the case of a mass spectrometry trace, a false positive is a detected peak that is not located within ± 1% of the *M*/*Z* value of the true peak. Supplementary Figure 3 shows a peak detection comparison of our PCWA with two other CWT-based methods. In Supplementary Fig. 3a, an example of a simulated protein spectrum is shown with gray vertical line markers, indicating the true location of peaks and colored markers are used

for the corresponding methods. PCWA detected 84% of peaks correctly (62% for Du et al and 86% for Zhang et al) with 1% FDR (32% for Du et al and 7% for Zhang et al). Supplementary Figure 3b shows the average of ROC curves for 75 randomly selected simulated protein mass spectra. The table in Supplementary Fig. 3c compares the average run time for typical size (~20,000 data points) of MS data files with 50 levels of logarithmic scale values. We observed that PCWA maintains excellent performance with significantly accelerated analysis time.

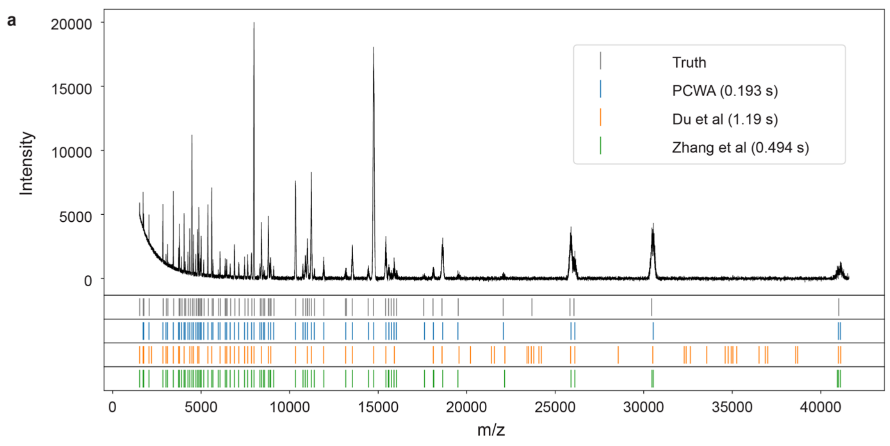

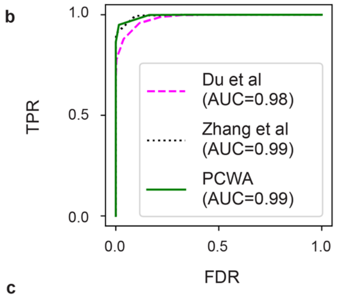

c

Method	Runtime [s]
Du et al	0.96 ±0.12
Zhang et al	0.44 ±0.04
PCWA	0.23 ±0.15

Supplementary Fig. 3 Mass spectroscopy peak detection. **a** An example of peak detection done by three CWT methods on a simulated protein spectrum. **b** ROC plot generated by varying the threshold (minimum SNR for Du et al and Zhang et al methods). Each spectrum has ~20,000 data points and 50 levels of logarithmic scale values are used to calculate CWT. **c** Run time comparison.

Multi-Spot Gaussian (MSG) Wavelet

Custom MSG wavelets are built up by adding *N* Gaussian functions representing the multi-spot excitation pattern in the analyte channels and encapsulating them with two negative Gaussian functions to provide a behavior similar to the Ricker wavelet. The side

negative peaks also ensure that standard wavelet requirements (zero mean and square norm of one) are fulfilled.

$$\psi_N(t,\Delta t) = \sum_{n=0}^{N-1} exp\left(\frac{-\left[t-\left(n-\frac{N-1}{2}\right)\Delta t\right]^2}{2\Delta t^2\sigma_+^2}\right)$$
$$-\sum_{k=\pm1}\frac{2a}{\Delta t\sigma_-}\phi\left(\frac{t+k\left(\sigma_- m_0-\frac{N}{2}\right)\Delta t}{\Delta t\sigma_-}\right)\Phi\left(k\alpha\frac{t+k\left(\sigma_- m_0-\frac{N}{2}\right)\Delta t}{\Delta t\sigma_-}\right)$$
$$\phi(t) = \frac{1}{\sqrt{2\pi}} exp\left(-\frac{t^2}{2}\right)$$
$$\Phi(t) = \int_{-\infty}^{t}\phi(u)du = \frac{1}{2}\left[1+erf\left(\frac{t}{\sqrt{2}}\right)\right]$$
$$m_0(\alpha) \approx \mu_z - \frac{\gamma_1\sigma_z}{2} - \frac{sgn(\alpha)}{2} exp\left(-\frac{2\pi}{|\alpha|}\right) \quad , \qquad (2)$$
$$\delta = \frac{\alpha}{\sqrt{1+\alpha^2}}$$
$$\mu_z = \sqrt{\frac{2}{\pi}}$$
$$\sigma_z = \sqrt{1-\mu_z^2}$$
$$\gamma_1 = \frac{4-\pi}{2}\frac{\left(\delta\sqrt{\frac{2}{\pi}}\right)^3}{\left(1-\frac{2\delta^2}{\pi}\right)^{3/2}}$$

Reference simulations for Multi-Peak Signal Analysis

The experimental single DNA data in the main manuscript do not consist of known events so that the accuracy of the different algorithms cannot be compared to a known ground truth. In order to eliminate this uncertainty, an additional benchmark analysis was done on a simulated multi-peak signal. The simulated trace was created by randomly placing 239 multi-peak events generated by 7 Gaussian functions. The width and the spacing between these Gaussian functions were carefully selected to replicate the experimental signals. Velocity and amplitude of each of these events were taken from the joint distribution of the actual events from the real tagged bacteria experiment with added noise. A constant dark current count plus scatterings from the buffer (here DI water) was

modeled and added to the signal. Finally, a Poisson function was applied to the resultant signal to reflect the SPCM photon detection error. Supplementary Figure 4 shows a side by side comparison of an example of the multi-peak signal from the KPC experiment with a simulated trace. (Note that the event frequency is higher in the simulated trace to limit the computational analysis time).

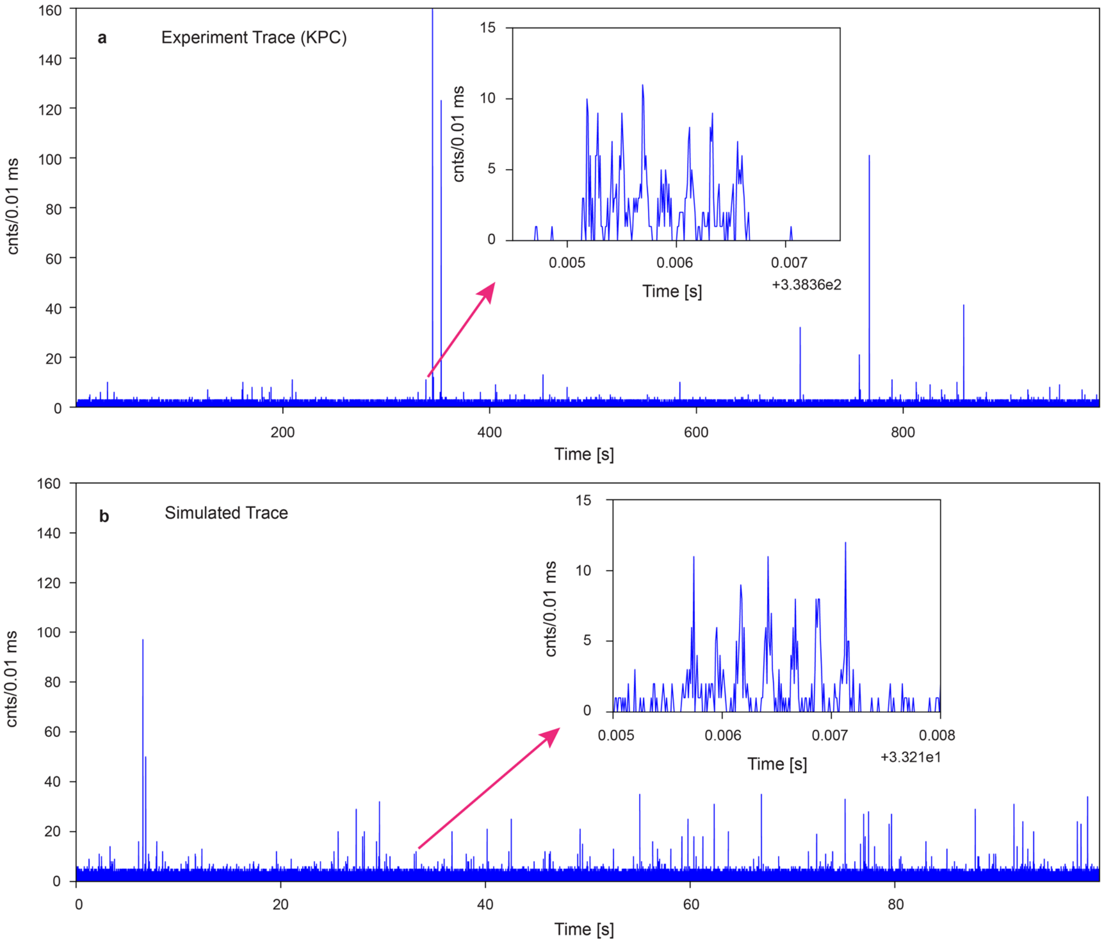

Supplementary Fig. 4: Experiment and simulated traces. **a** The fluorescence signal recorded from the KPC detection experiment (Fig. 4, main manuscript). Inset shows a randomly selected event. **b** The simulated trace with similar joint amplitude-velocity distribution with added noise. Inset shows a zoomed in window of one of the 239 events.

The same three methods were then used for detection and identification of multi-peak events and the results are summarized in Supplementary Table 1. The PCWA (MSG) algorithm is able to detect almost 5x more events than the Shift-Multiply method with only 0.4% of them located incorrectly. Lowering the threshold in Shift-Multiply results in a drastic drop of accuracy and increase in false discovery rate. An over 2x lower false identification rate of events was observed for PCWA (MSG) compared to the Shift-Multiply.

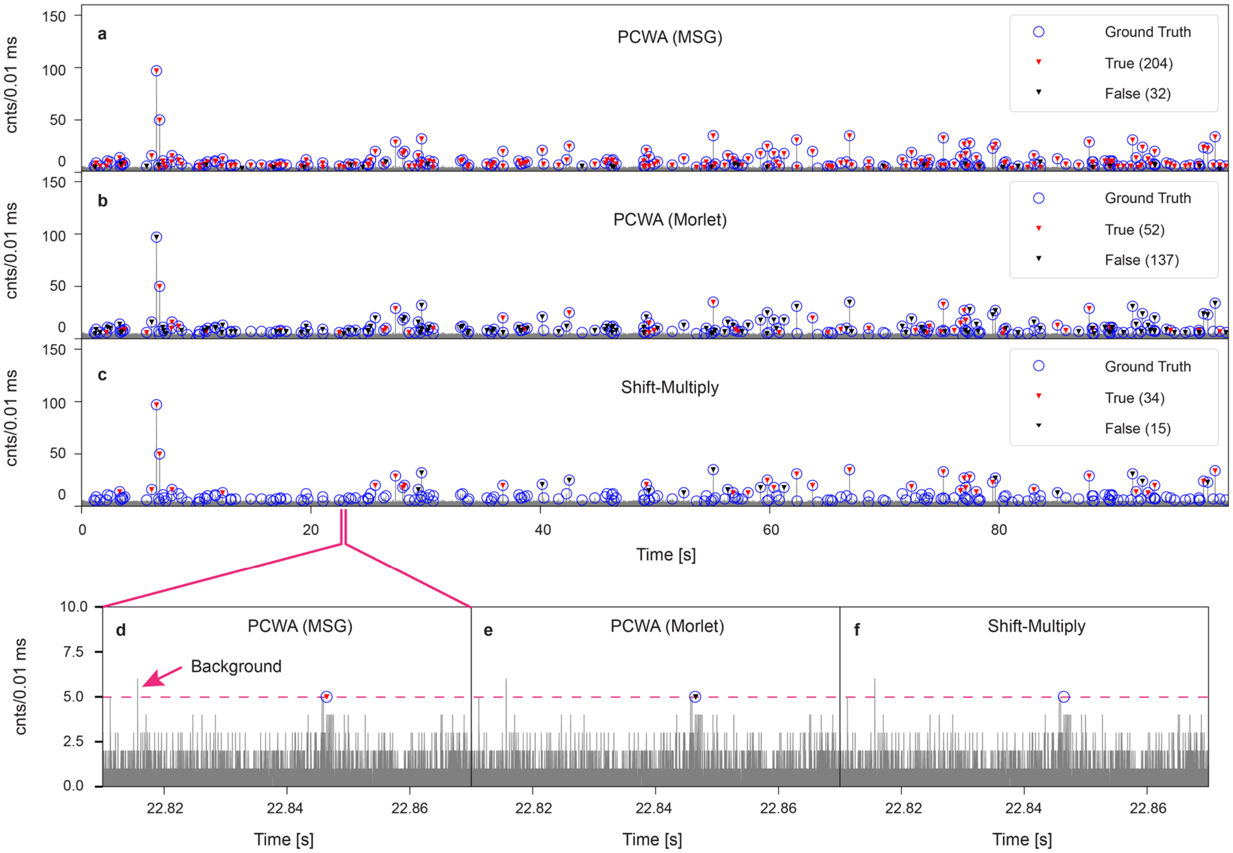

Supplementary Fig. 5 Performance comparison of three methods on simulated trace. **a** Events detected using PCWA with MSG wavelet. Most of the events (>98%), even those buried in the background, were accurately identified (86%). Only one false event from noise was picked as an event. **b** Detection done by PCWA with Morlet wavelet. Compared to MSG wavelets, significantly less accurate localization of events with more FDR is observed. The accuracy of identifying the type of target is the worst when using

Morlet wavelet. **c** Detected events using Shift-Multiply technique. Comparable background level to weak events prevents accurately locating and identifying events. **d-f** An example of a weak signal analyzed by three methods; only PCWA (MSG) is able to precisely locate and identify it.

PCWA with Morlet wavelet is able to pick a high rate of event but lacks the accuracy in identifying the number of peaks provided by the MSG wavelet. The acceptable error in locating events is 7 ms from the ground truth location.

	**Shift-Multiply**	**PCWA (Morlet)**	**PCWA (MSG)**
**Total Detected**	49	189	236
**TPR**	0.205	0.782	0.983
**FDR**	0.000	0.011	0.004
**Accuracy [%]**	69.4	27.5	86.4

Supplementary Table 1 Performance comparison of Shift-Multiply and PCWA methods with simulated fluorescence signal. The metrics are measured based on the ground truth event list.

Supplementary References

1. Deamer, D., Akeson, M. & Branton, D. Three decades of nanopore sequencing. *Nat. Biotechnol.* **34**, 518–524 (2016).

2. Venkatesan, B. M. & Bashir, R. Nanopore sensors for nucleic acid analysis. *Nat. Nanotechnol.* **6**, 615–624 (2011).
3. Clarke, J. *et al.* Continuous base identification for single-molecule nanopore DNA sequencing. *Nat. Nanotechnol.* **4**, 265–270 (2009).
4. Rahman, M. *et al.* Optical trapping assisted detection rate enhancement of single molecules on a nanopore optofluidic chip. *Optica* **6**, 1130–1131 (2019).
5. Rahman, M. *et al.* On demand delivery and analysis of single molecules on a programmable nanopore-optofluidic device. *Nat. Commun.* **10**, 3712 (2019).
6. Du, P., Kibbe, W. A. & Lin, S. M. Improved peak detection in mass spectrum by incorporating continuous wavelet transform-based pattern matching. *Bioinformatics* **22**, 2059–2065 (2006).
7. Zhang, Z.-M. *et al.* Multiscale peak detection in wavelet space. *Analyst* **140**, 7955–7964 (2015).
8. Morris, J. S., Coombes, K. R., Koomen, J., Baggerly, K. A. & Kobayashi, R. Feature extraction and quantification for mass spectrometry in biomedical applications using the mean spectrum. *Bioinforma. Oxf. Engl.* **21**, 1764–1775 (2005).
